# Acceptance of illness and quality of life of patients under long-term home nursing care

**DOI:** 10.3389/fpubh.2025.1505164

**Published:** 2025-03-31

**Authors:** Bożena Majchrowicz, Krystyna Kowalczuk, Katarzyna Tomaszewska

**Affiliations:** ^1^Department of Nursing, Institute of Health Protection, State Academy of Applied Sciences, Przemyśl, Poland; ^2^Department of Integrated Medical Care, Medical University of Białystok, Białystok, Poland; ^3^Department of Nursing, Institute of Health Protection, The Bronisław Markiewicz Academy of Applied Sciences, Jarosław, Poland

**Keywords:** long-term home nursing care, patient, functional capacity, illness acceptance, life satisfaction

## Abstract

**Introduction:**

Long-term home nursing care is care for patients who do not qualify for inpatient treatment, and for various reasons are unable or unwilling to receive care in long-term facilities. Patients receiving such care are of various ages, with varying degrees of disabilities that limit their independent functioning. Their condition is caused by chronic diseases, traffic accidents or genetic diseases. In many cases, in a short period of time they turn from being professionally, socially active people to becoming dependent on third parties. Acceptance of one’s own illness can reduce the negative feelings associated with it, allows one to maintain self-esteem and is of great importance for the subjective feeling of life satisfaction.

**Aim:**

The aim of this study was to demonstrate whether a relationship exists among respondents receiving long-term home nursing care between the level of functional capacity, acceptance of illness and subjective assessment of life satisfaction.

**Materials and methods:**

The authors conducted a study among 240 patients under long-term home nursing care in Subcarpathian Voivodeship in Poland. The study used a diagnostic survey as a survey technique. The research tool was a survey questionnaire containing questions on sociodemographic data and standardized research tools: Barthel Scale, Acceptance of Illness Scale (AIS) and Satisfaction with Life Scale (SWLS). Mann–Whitney U test and Spearman’s rho coefficient were used in the statistical analysis. Statistical significance of *p* ≤ 0.05 was assumed.

**Results:**

The average illness acceptance score determined by respondents according to the AIS scale was 16.11 ± 6.57. The minimum level of illness acceptance in the study group was 8 pts., while the maximum was 40 pts. In the course of the analyses, it turned out that only in the group of patients over 65 years of age, life satisfaction increased as the level of illness acceptance increased. The correlation coefficient was statistically significant (*p* < 0.001) and showed a clear strength of association (*Spearman’s rho* = 0.450). In addition, with greater functional capacity, greater life satisfaction can be observed, but in this case, although the correlation was statistically significant (*p* < 0.05) it is characterized by a weak strength of the relationship (*Spearman’s rho* = 0.178).

**Conclusion:**

The age of respondents has an impact on life satisfaction of the respondents under long-term home care. The younger the patients, the lower the acceptance of the illness and the worse the evaluation of subjective quality of life. The respondents’ level of independence and the duration of long-term care coverage have a positive effect on the acceptance of the illness and the respondents’ subjective assessment of life satisfaction.

## 1 Introduction

The continued growth of the older adult in any population and the lack of multi-generational families are among the many reasons for the need to develop long-term care. According to the WHO definition, long-term care is „a system of activities undertaken by informal (family, friends, neighbors) and/or formal (medical, social) caregivers to ensure that a person who is unable to perform self-care activities maintains the highest quality of life, in accordance with personal preferences and requirements, with the highest possible level of independence, autonomy, participation and personal dignity.” It is believed that the combination of nursing and care services into a single complex coordinated by a nurse specialist provides an opportunity for the development of quality long-term care (1). The complexity of the occurrence of functional changes in chronically ill people and the deficit of self-care and self-care causes them to benefit from various forms of home care (2).

Patients who do not require hospital treatment, but due to health problems require systematic nursing care, are qualified for long-term home care provided by nurses. Long-term home nursing care in Poland is a guaranteed care service, financed by universal health insurance premiums under contracts with the National Health Fund (NFZ), the program has been in existence since 2004 (3). Persons requiring assistance in performing activities of daily living used to be defined as unable to live independently, and nowadays the concept of dependency resulting from damage or impairment of bodily functions that creates the need for constant or long-term care or assistance in performing basic activities of daily living is increasingly used to describe this condition (4). The provider of long-term home care is a nurse who intervenes in nursing, educational, rehabilitative or diagnostic activities. The type and scope of activities undertaken depends mainly on the functional capacity of the patient. Functional deficits concerning both the biological and psychosocial spheres are diagnosed in residents receiving long-term home care (1). Barthel scale is used for assessment of the patient—if 40 points or less is obtained, it means that the patient is qualified for long-term care as one requires constant care.

Functioning with chronic illness poses enormous challenges for patients because it interferes with various bodily functions: physical, mental and social; and thus affecting their quality of life. Chronically ill patients face serious problems such as higher medical expenses, social isolation and loneliness, disability, fatigue, pain, discomfort, feelings of anxiety, anger, hopelessness, frustration, fear, and depression (5).

Effectiveness in coping with chronic illness depends on the type of illness, the patient’s personal resources, and the influence of the physical and social environment (6). Acceptance of one’s own illness can affect the reduction of negative feelings associated with it, reduce stress caused by deterioration of health, and allows to maintain self-esteem. This is of great importance for the subjective perception of quality of life by the person receiving long-term care and the level of his own activity in all spheres (7). It becomes very important to accept the disease, which makes it easier for everyone to function daily in the new reality, to adapt to life with a disability. The concept of disease acceptance is understood as coming to terms with the fact that one is ill and recognizing the need to adapt to the disease and its consequences. Greater acceptance of illness has a positive effect on self-management of health and is associated with a better quality of life for patients (8–10). A person’s quality of life, including both lower-and higher-order aspects, results from a range of activities and experiences, many of which can potentially be affected by care services, depending on the nature of the care tasks undertaken (11). Assessing life satisfaction and acceptance of illness helps identify patients’ real problems and needs, and is one of the most difficult stages of the illness process. It is believed that the higher it is, the better the adaptation and the less psychological discomfort. Reduced levels of acceptance can affect the overall level of satisfaction with life, which is the result of comparing one’s own situation with self-established standards (12).

Numerous studies have identified predictors of life satisfaction among the older adult, such as housing conditions and social support, which can be important factors in life satisfaction (13–18). Many researchers have argued that there is also a relationship between functional capacity and life satisfaction among older people receiving long-term care (19, 20). Most of the published studies are concerned with life satisfaction of people receiving inpatient care, lacking in the area of nursing home long-term care. Therefore, the authors took as an aim of the study to investigate whether in the surveyed group of patients receiving long-term home nursing care, there is a relationship between the level of functional capacity, acceptance of the disease and subjective assessment of life satisfaction. Based on the aim of the study, the following objectives were formulated:

1. What is level of functional fitness of respondents under long-term home nursing care?
2. Does the respondents’ functional fitness affect acceptance of chronic disease?
3. Does life satisfaction depend on the age, acceptance of the disease and functional fitness of the respondents?

## 2 Materials and methods

### 2.1 Research design

In the present study, a survey was conducted among 240 patients under long-term home nursing care in Subcarpathian Voivodeship in Poland. A diagnostic survey method was used for measurements. The research tool was a survey questionnaire containing questions on sociodemographic data and standardized questionnaires: *Barthel Scale*, *Acceptance of Illness Scale (AIS)* and *Satisfaction with Life Scale (SWLS)*. The survey was conducted between November 2023 and February 2024.

### 2.2 Research tools

#### 2.2.1 Barthel Scale

The Barthel Scale is used to assess basic activities of daily living. Depending on the extent of independence, the patient is given between 0 and 100 points. The number of points obtained indicates the degree of dexterity deficit and determines his condition and need for care. We evaluated the usefulness of the Barthel questionnaire in the Polish healthcare setting as a reliable tool (*α*-Cronbach’s coefficient = 0.78 ± 0.89; test–retest correlation coefficient R = 0.93/0.95) for measuring the extent of independence in performing activities of daily living, which was confirmed in a study (21). Patients who scored between 0 and 40 points on the Barthel scale are eligible for care.

#### 2.2.2 Acceptance of Illness Scale

The Acceptance of Illness Scale (AIS), created by Felton et al. of the Center Community Research and Action, Department of Psychology, New York University was used in a Polish adaptation by Juczynski (22). The scale is used to determine the level of acceptance of illness among sick people. It uses eight statements assessing the limitations associated with the disease. Their main objective is recognition of the limitations imposed by the illness, lack of self-sufficiency, a sense of dependence on others and lowered self-esteem. Respondents are given the opportunity to select a response on a scale of 1 to 5, and depending on the number of points obtained, the level of acceptance is assessed—the higher the score, the greater the acceptance and the patient has fewer negative feelings about the disease process. The Cronbach’s alpha internal consistency index was 0.85, with satisfactory constancy of the score at 0.64. The results of the AIS Scale also correlated with the results of other tools that indirectly inform about the acceptance of the disease by different groups of patients (multiple sclerosis, diabetics and people after myocardial infarction). It can be applied to any disease. The greater the acceptance of the disease, the better the adaptation and less psychological discomfort.

#### 2.2.3 Satisfaction with Life Scale

Satisfaction with Life Scale (SWLS) by Ed Diener, Robert A. Emmons, Randy J. Larsen, Sharon Grioffin was adapted by Zygfryd Juczynski (22). The scale assesses the level of the overall life satisfaction index. The respondent determines to what extent each of the questionnaire’s statements applies to his current life situation. Patient can score 5–35 points and the higher the score, the greater the sense of satisfaction with life. The Cronbach’s alpha reliability index of the SWLS, determined in a survey of 371 people, was found to be satisfactory (0.81). The scale’s coefficient of constancy, established in a two-study of a group of 30 people 6 weeks apart, was 0.86. Theoretical accuracy was estimated by analyzing associations with variables that indirectly reflect or influence feelings of life satisfaction.

### 2.3 Participants

The study included 240 patients receiving long-term home nursing care in southeastern Poland. Respondents who were able to consent to participate in the study were included. This means that communication and home care coverage were possible in all the cases. The authors hand-delivered the prepared paper survey forms to 5 facilities providing home care services to patients. At a meeting with nursing staff, information was provided on the purpose of the study, how to complete the questionnaire with a request to conduct the survey in the home setting. 350 questionnaires were distributed, and after 2 months, 240 correctly filled out were received (68.6%).

### 2.4 Ethical procedure

The application was favorably approved by the Bioethics Committee of the State Academy of Applied Sciences in Przemyśl (KBPANS 2/2024).

### 2.5 Statistical analysis

Mann Whitney-U tests and Spearman’s rho coefficient were used in the statistical analysis. Statistical significance of *p* ≤ 0.05 was assumed.

## 3 Results

The study included 240 patients under long-term home nursing care. Sociodemographic data are included in Table 1.

**Table 1:** Characteristics of the study group.

Variable		Frequency (%)	Percentage (%)
Gender	Female	156	65.0
	Male	84	35.0
Age (years)	18–25	2	0.8
	26–35	11	4.6
	36–45	12	5.0
	46–55	21	8.8
	56–65	36	15.0
	Above 65	158	65.9
Place of residence	City	104	43.3
	Village	130	54.2
Duration of care (years)	Below 1	42	17.5
	1–5	128	53.3
	6–10	45	18.8
	Above 10	22	9.2
Who cares for the patient besides the long-term home care nurse?	Immediate family (husband/wife, parents, siblings, children)	175	71.1%
	Extended family	12	4.9%
	Neighbors	6	2.4%
	Social assistance	33	13.4%
	Single	20	8.1%

When asked about their primary disease entity, respondents indicated as many as 31 different diseases that had been diagnosed in them and warranted long-term home nursing care, due to functional deficits. In younger people, these included post-accident spinal injury 3.2%, spinal muscular atrophy (1.7%) or multiple sclerosis 5.0%. Older people had senile dementia (14.4%), Alzheimer’s disease (5.0%), had suffered a stroke (22.0%), had complications of diabetes—diabetic foot (5.5%) or ulcers of the lower extremities (4.6%). They also had other comorbidities that reduced their functional capacity.

The mean level of acceptance of illness (AIS) in the surveyed group of long-term care patients was 16.11 ± 6.57. The minimum level of acceptance of illness among the respondents was 8 pts., while the maximum was 40 pts. (Table 2).

**Table 2:** Mean level of disease acceptance, life satisfaction, and functional capacity.

Variable	AIS disease acceptance level (8–40)	Satisfaction with life SWLS (5–35)	Barthel functional ability (0–40)
Frequency (*n*)	240	240	240
Mean	16.11	18.62	22.61
Median	15.50	19.00	25.00
Standard deviation	6.57	6.06	12.36
Minimum	8	5	0
Maximum	40	33	40

Statistical analysis showed that the level of acceptance of illness (AIS), satisfaction with life (SWLS) and functional capacity assessed according to the Barthel scale were not statistically significantly differentiated by gender and place of residence of home care respondents (*p* > 0.05).

A higher level of functional ability as assessed by the Barthel scale is associated with a higher level of acceptance of the disease (AIS). The relationship is statistically significant (*p* < 0.001) at the level of *Spearman’s rho* = 0.267 (Table 3).

**Table 3:** Relationship between level of acceptance of illness and functional performance.

Variable			Barthel functional ability (0–40)
Spearman’s rho	AIS disease acceptance level (8–40)	Correlation coefficient	0.267
		Frequency (*n*)	240

The study showed no relationship between functional capacity, level of disease acceptance and life satisfaction (*p* > 0.05; Table 4).

**Table 4:** Relationship between respondents’ life satisfaction and level of acceptance of illness and functional capacity.

Variable			Barthel functional ability (0–40)	AIS disease acceptance level (8–40)
Spearman’s rho	Satisfaction with life SWLS (5–35)	Correlation coefficient	0.118	0.354
		Frequency (*n*)	240	240

In each age group, which had a size of more than five patients, calculations were made to see if there were statistically significant correlations (*p* < 0.05) between functional capacity, level of acceptance of the disease and life satisfaction. During the analyses, it was found that only in the group of patients over 65 years of age, life satisfaction increases as the level of disease acceptance increases. The correlation coefficient is statistically significant (*p* < 0.001) and has a clear strength of association (*Spearman’s rho* = 0.450). In addition, a higher level of life satisfaction can be observed with higher functional capacity, but in this case, although the correlation is statistically significant (*p* < 0.05) it is characterized by a weak strength of the relationship (*Spearman’s rho* = 0.178; Table 5).

**Table 5:** Relationship between age of respondents and level of acceptance of illness, functional ability and life satisfaction.

Age = 36–45 years			Barthel functional ability (0–40)	AIS disease acceptance level (8–40)
Spearman’s rho	Satisfaction with life SWLS (5–35)	Correlation coefficient	−0.296	0.600
		Significance (two-tailed)	0.518	0.154
		Frequency (*n*)	7	7
Age = 46–55 years			Barthel functional ability (0–40)	AIS disease acceptance level (8–40)
Spearman’s rho	Satisfaction with life SWLS (5–35)	Correlation coefficient	−0.342	−0.050
		Significance (two-tailed)	0.130	0.831
		Frequency (*n*)	21	21
Age = 56–65 years			Barthel functional ability (0–40)	AIS disease acceptance level (8–40)
Spearman’s rho	Satisfaction with life SWLS (5–35)	Correlation coefficient	0.012	0.008
		Significance (two-tailed)	0.946	0.966
		Frequency (*n*)	35	35
Age = Above 65 years			Barthel functional ability (0–40)	AIS disease acceptance level (8–40)
Spearman’s rho	Satisfaction with life SWLS (5–35)	Correlation coefficient	0.178	0.450
		Significance (two-tailed)	0.027	0.000
		Frequency (*n*)	154	154

Similarly, in the four categories of time in care, the possible existence of statistically significant (*p* < 0.05) relationships between functional fitness, level of acceptance of the disease and life satisfaction was checked. When the time of care is less than 1 year, life satisfaction increases as the level of acceptance of the disease and functional fitness increases. The correlation coefficients are statistically significant (*p* < 0.05) and exhibit moderate strengths of association. In addition, during care coverage from 1 to 5 years with higher levels of disease acceptance, life satisfaction increases. The correlation is statistically significant (*p* < 0.01) and characterized by moderate strength of association (*Spearman’s rho* = 0.301). There was no statistically significant correlation between functional capacity and life satisfaction (*p* > 0.05). Considering the other categories of time of care coverage, there is no statistically significant correlation between the analyzed variables (Table 6).

**Table 6:** Relationship between respondents’ time in care and level of acceptance of illness, functional ability and life satisfaction.

Duration of care—below 1 year			Barthel functional ability (0–40)	AIS disease acceptance level (8–40)
Spearman’s rho	Satisfaction with life SWLS (5–35)	Correlation coefficient	0.329	0.383
		Significance (two-tailed)	0.033	0.012
		Frequency (*n*)	42	42
Duration of care—1–5 years			Barthel functional ability (0–40)	AIS disease acceptance level (8–40)
Spearman’s rho	Satisfaction with life SWLS (5–35)	Correlation coefficient	0.130	0.301
		Significance (two-tailed)	0.148	0.001
		Frequency (*n*)	126	126
Duration of care—6–10 years			Barthel functional ability (0–40)	AIS disease acceptance level (8–40)
Spearman’s rho	Satisfaction with life SWLS (5–35)	Correlation coefficient	−0.082	0.250
		Significance (two-tailed)	0.609	0.115
		Frequency (*n*)	41	41
Duration of care—Above 10 years			Barthel functional ability (0–40)	AIS disease acceptance level (8–40)
Spearman’s rho	Satisfaction with life SWLS (5–35)	Correlation coefficient	0.235	0.487
		Significance (two-tailed)	0.400	0.066
		Frequency (*n*)	15	15

Older adult patients have a higher level of fitness; moreover, with longer duration of care, the level of acceptance of the disease increases. The correlation coefficients are statistically significant (*p* < 0.05) but the strengths of the relationship as determined by Spearman’s rho coefficient were found to be insignificant (Table 7).

**Table 7:** Relationship between age and time of care coverage of respondents and level of acceptance of illness, functional ability, and life satisfaction.

			Age	Duration of care
Spearman’s rho	Barthel functional ability (0–40)	Correlation coefficient	0.217	−0.096
		Significance (two-tailed)	0.001	0.142
		Frequency (*n*)	240	240
	AIS disease acceptance level (8–40)	Correlation coefficient	−0.013	0.136
		Significance (two-tailed)	0.846	0.042
		Frequency (*n*)	240	240
	Satisfaction with life SWLS (5–35)	Correlation coefficient	0.029	0.038
		Significance (two-tailed)	0.663	0.567
		Frequency (*n*)	240	240

## 4 Discussion

The purpose of this study was to demonstrate whether a relationship exists between the level of functional capacity, acceptance of illness and subjective assessment of life satisfaction among a surveyed group of patients receiving long-term home nursing care. Reviewing the literature, it was found that researchers rarely address the issue of disease acceptance and quality of life for patients receiving long-term home care. There are almost exclusively analyses relating to patients who receive this care, but within nursing and treatment institutions.

In the course of analyses conducted in the present study, it was found that only in the group of patients over the age of 65 did an increase in disease acceptance correlate with a higher level of life satisfaction. In the younger group, these relationships were not statistically significant. Kowalska et al. demonstrated that there is a correlation between the degree of disease acceptance and the functional capacity of older adult respondents. The poorer the initial functional status, the lower the level of disease acceptance (7), a finding that is confirmed by other studies (23).

Jankowska-Polanska et al. (24) also found that disease acceptance has a significant impact on patients’ quality of life scores—the higher the acceptance, the higher the quality of life score. By accepting one’s chronic disease, the patient adapts to it more easily, which can improve one’s overall quality of life and reduce one’s hospital stays (25, 26). In the present study, the levels of disease acceptance, life satisfaction, and functional capacity did not differ significantly based on gender or place of residence among respondents receiving home care. Other authors also have shown that the level of functional performance of the subjects was not dependent on sociodemographic factors and the time of coverage of long-term home care. The level of functional performance of the subjects was influenced by the type of chronic disease they had (18).

A study of patients receiving long-term home care for mechanically ventilated patients using non-invasive and invasive methods provides insight into the impact of the treatment method used on patients’ level of disease acceptance and life satisfaction. The study showed that the level of disease acceptance and life satisfaction among the patients surveyed was at an average level. Statistical analysis showed that higher levels of disease acceptance were associated with higher life satisfaction (6). In studies conducted in a residential long-term care facility, the obtained health status results were directly proportional to the subjective assessment of quality of life (27). Another study analyzed the relationship between fitness and environmental factors and life satisfaction showed that the effect of functional independence on life satisfaction was not significant. Support from the environment was also important for respondents to improve life satisfaction for older people with care needs. The results of this study suggest that in order to maintain and improve life satisfaction among older adults with care needs, it is important to focus on environmental factors and support them to promote participation in desired activities, rather than improving their functional independence (28). Respondents in a study of patients with rheumatoid arthritis during disease exacerbation reported low and moderate levels of life satisfaction, patients in remission had moderate results, while those in the control group described their level of life satisfaction as high and moderate (29).

Kieltyka et al. conducted a research to compare selected aspects of quality of life in older adult people with chronic illnesses who are in institutional care and those who remain in their own homes. The authors proved that respondents of treatment and care facilities rated lower satisfaction with their quality of life and their health compared to seniors staying at home under family care. Significantly better quality of life in four domains was found in the group of seniors staying in their own homes compared to the wards of a care facility (30). It can be concluded that the environment in which patients reside may be crucial to their sense of quality of life.

In the present study, among younger individuals with a shorter duration of care, an increase in disease acceptance and functional capacity was associated with higher life satisfaction. A significant predictor was the desire to regain full functional capacity and return to previously held social roles.

As already mentioned, most of the publications addressing the problems of chronically ill people receiving home care address only selected aspects, most often functional impairment. A recent Canadian study found that between 23% and 54% of home care clients have unmet functional and supportive care needs (31). Expanding care to include needs that maintain independence, such as access to assistive technology and removal of architectural barriers, can significantly improve disease acceptance and life satisfaction (32).

## 5 Conclusion

The results proved that the functional fitness of the respondents depends on their age, older respondents have better fitness. The respondents’ higher level of fitness influences greater acceptance of the disease as well as an increase in their life satisfaction. The age of respondents has an impact on the quality of life of long-term care patients surveyed. The younger the patients, the lower the acceptance of the disease and the worse they evaluate their subjective quality of life. The respondents’ level of independence and the duration of long-term care coverage affect the acceptance of the disease and the respondents’ subjective assessment of their quality of life.

## 6 Limitations of the study

The results of the conducted study are based on solid foundations; however, certain limitations should be acknowledged. The study focused on a group of patients receiving home-based long-term nursing care, encompassing individuals of varying ages and with different medical conditions. Due to the considerable diversity of these conditions, they were not included in the present work, which may limit the significance of comparisons between different populations. Individual responses to chronic illnesses and the circumstances necessitating long-term care vary, making such comparisons potentially less reliable.

Additionally, the small sample size may restrict the generalizability of the findings. Further multi-center studies are necessary to enhance the applicability of the results. Methodological limitations also include the cross-sectional design of the study, which prevents the identification of causal relationships between variables. Furthermore, reliance on self-assessment questionnaires, the voluntary nature of participation, and the unknown reasons for non-returned surveys may affect the representativeness of the sample.

Results can be cautiously generalized to patients in other regions or countries with some limitations. Factors such as regional health care infrastructure, local support systems and cultural attitudes toward long-term care may affect the results. Expanding the study to multiple regions or countries with diverse health care systems will provide more robust and generalizable results.

## Data Availability

The raw data supporting the conclusions of this article will be made available by the authors, without undue reservation.

## References

[1] Bartoszek A Kocka K Rząca M Nowicki G Ślusarska B. Range and frequency of nursing interventions undertaken in patients with functional performance deficits in home care. Long Term Care Nurs. (2020) 5:131–46. doi: 10.19251/pwod/2020.2(4)

[2] Dhamanti I Nia IM Nishara K Srikaanth BP. Smart home healthcare for chronic disease management: a scoping review. Digit Health. (2023) 9:20552076231218144. doi: 10.1177/20552076231218144, PMID: 38074339 PMC10702417

[3] Ziembicka DM Lukaszuk B Marcinowicz L. Evaluation of the functioning of long-term at-home nursing care in Poland from the perspective of care providers: mixed methods study. J Clin Nurs. (2023) 32:485–93. doi: 10.1111/jocn.16255, PMID: 35225374

[4] Kilian J Ćwirlej-Sozańska A Wiśniowska-Szurlej A Wilmowska-Pietruszyńska A. System długoterminowej opieki domowej nad osobami starszymi w Polsce i wybranych krajach europejskich. Niepełnosprawność zagadnienia problemy rozwiązania. (2018) 4:113.

[5] Van Wilder L Pype P Mertens F Rammant E Clays E Devleesschauwer B. Living with a chronic disease: insights from patients with a low socioeconomic status. BMC Fam Pract. (2021) 22:233. doi: 10.1186/s12875-021-01578-7, PMID: 34789153 PMC8598397

[6] Tomaszewska K Majchrowicz B Kowalczuk K. Acceptance of illness and life satisfaction in mechanically ventilated patients depending on the type of disease, type of ventilation and demographic factors. Long Term Care Nurs. (2023) 8:3–15.

[7] Kowalska J Wolny K Kobylańska M Wójcik B. The degree of acceptance of illness and functional status among elderly people staying in the rehabilitation Centre. Geriatria. (2015) 9:3–9.

[8] Juzwiszyn J Łabuń A Tański W Szymańska-Chabowska A Zielińska D Chabowski M. Acceptance of illness, quality of life and nutritional status of patients after lower limb amputation due to diabetes mellitus. Ann Vasc Surg. (2022) 79:208–15. doi: 10.1016/j.avsg.2021.07.023, PMID: 34644635

[9] Szcześniak M Świątek AH Cieślak M Świdurska D. Disease acceptance and eudemonic well-being among adults with physical disabilities: the mediator effect of meaning in life. Front Psychol. (2020) 11:525560. doi: 10.3389/fpsyg.2020.525560, PMID: 33192766 PMC7643024

[10] Qiu C Zhang X Zang X Zhao Y. Akceptacja choroby pośredniczy w efektach umiejętności czytania i pisania w zakresie zdrowia w zachowaniach samokontroli. Eur J Cardiovasc Nurs. (2020) 19:411–20. doi: 10.1177/1474515119885240, PMID: 31722553

[11] Syah R Yetti K Novieastari E Gayatri D Nining S. Factors influencing patient satisfaction in home care services: A systematic review. F1000Res. (2024) 13:969. doi: 10.12688/f1000research.154937.1

[12] Hamerlińska A Kamyk-Wawryszuk A. Akceptacja choroby i satysfakcja z życia dorosłych osób z chorobami rzadkimi. Acceptance of the disease and satisfaction with life of adults with rare diseases. Niepełnosprawność-Dyskursy Pedagogiki Specjalnej. (2022) 44:42–53.

[13] Benkel I Arnby M Molander U. Living with a chronic disease: a quantitative study of the views of patients with a chronic disease on the change in their life situation. SAGE Open Med. (2020) 8:2050312120910350. doi: 10.1177/2050312120910350 32341782 PMC7171994

[14] Mekonnen HS Lindgren H Geda B Azale T Erlandsson K. Satisfaction with life and associated factors among elderly people living in two cities in Northwest Ethiopia: a community-based cross-sectional study. BMJ Open. (2022) 12:e061931. doi: 10.1136/bmjopen-2022-061931, PMID: 36581991 PMC9438199

[15] Papi S Cheraghi M. Multiple factors associated with life satisfaction in older adults. Prz Menopauzalny. (2021) 20:65–71. doi: 10.5114/pm.2021.107025, PMID: 34321983 PMC8297631

[16] Lin Y Xiao H Lan X Wen S Bao S. Living arrangements and life satisfaction: mediation by social support and meaning in life. BMC Geriatr. (2020) 20:136. doi: 10.1186/s12877-020-01541-8, PMID: 32293305 PMC7158054

[17] Qazi SL Koivumaa-Honkanen H Rikkonen T Sund R Kröger H Isanejad M. Physical capacity, subjective health, and life satisfaction in older women: a 10-year follow-up study. BMC Geriatr. (2021) 21:658. Published 2021. doi: 10.1186/s12877-021-02605-z, PMID: 34814850 PMC8609741

[18] Liu LH Kao CC Ying JC. Functional capacity and life satisfaction in older adult residents living in long-term care facilities: the mediator of autonomy. J Nurs Res. (2020) 28:e102. doi: 10.1097/JNR.0000000000000362, PMID: 31904735

[19] Tian H Chen J. Study on life satisfaction of the elderly based on healthy aging. J Healthc Eng. (2022) 2022:8343452–7. doi: 10.1155/2022/8343452, PMID: 36226197 PMC9550455

[20] Buckinx F Peyrusqué E Kergoat MJ Aubertin-Leheudre M. Reference standard for the measurement of loss of autonomy and functional capacities in long-term care facilities. J Frailty Aging. (2023) 12:236–43. doi: 10.14283/jfa.2023.4, PMID: 37493385

[21] Kuźmicz I Brzostek T Górkiewicz M. Kwestionariusz Barthel jako narzędzie pomiaru zakresu samodzielności fizycznej osób w podeszłym wieku. Stud Med. (2008) 12:17–21.

[22] Juczyński Z. Narzędzia pomiaru w promocji zdrowia. Warszawa: Pracownia testów Psychologicznych Polskiego Towarzystwa Psychologicznego (2001).

[23] Uchmanowicz I Jankowska-Polanska B Motowidlo U Uchmanowicz B Chabowski M. Assessment of illness acceptance by patients with COPD and the prevalence of depression and anxiety in COPD. Int J Chron Obstruct Pulmon Dis. (2016) 11:963–70. doi: 10.2147/COPD.S102754, PMID: 27274217 PMC4869633

[24] Jankowska-Polańska B Kasprzyk M Chudiak A Uchmanowicz I. Effect of disease acceptance on quality of life in patients with chronic obstructive pulmonary disease (COPD). Pneumonol Alergol Pol. (2016) 84:3–10. doi: 10.5603/PiAP.a2015.0079, PMID: 26687669

[25] Molina-Mula J Miguélez-Chamorro A Taltavull-Aparicio JM Miralles-Xamena J Ortego-Mate MDC. Quality of life and dependence degree of chronic patients in a chronicity care model. Healthcare. (2020) 8:293. doi: 10.3390/healthcare8030293, PMID: 32846995 PMC7551615

[26] Wysocki G Czapla M Uchmanowicz B Fehler P Aleksandrowicz K Rypicz Ł. Influence of disease acceptance on the quality of life of patients with ankylosing spondylitis-single Centre study. Patient Prefer Adherence. (2023) 17:1075–92. doi: 10.2147/PPA.S403437, PMID: 37090183 PMC10119491

[27] Witsø AE Eide AH Vik K. Older homecare service recipients’ satisfaction with participation in daily life activities. Phys Occup Therapy Geriatr. (2012) 30:85–101. doi: 10.3109/02703181.2012.678970

[28] Misu Y Hayashi S Iwai N Yamamoto T. Factors affecting the life satisfaction of older people with care needs who live at home. Geriatrics. (2022) 7:117. doi: 10.3390/geriatrics7050117, PMID: 36286220 PMC9601634

[29] Staszkiewicz M Kulesa-Mrowiecka M Szklarczyk J Jaworek J. Life satisfaction, generalized sense of self-efficacy and acceptance of illness in rheumatoid arthritis patients depending on age and severity of the disease. Reumatologia. (2023) 61:175–85. doi: 10.5114/reum/168294, PMID: 37522147 PMC10373164

[30] Kiełtyka A Leja M Lubińska-Żądło B Kiełtyka B. Ocena jakości życia osób w wieku podeszłym ze schorzeniami przewlekłymi. Praca Socjalna. (2017) 5:81–103.

[31] Zuverink A Xiang X. Anxiety and unmet needs for assistance with daily activities among older adults. J Aging Health. (2020) 32:491–500. doi: 10.1177/0898264319830805, PMID: 30832547

[32] Saari ME Giosa JL Holyoke P Heckman GA Hirdes JP. Profiling the medical, functional, cognitive, and psychosocial care needs of adults assessed for home care in Ontario, Canada: the case for long-term 'life care' at home. PLoS One. (2024) 19:e0300521. doi: 10.1371/journal.pone.0300521, PMID: 38558082 PMC10984553

